# Electrochemical Behavior of Inductively Sintered Al/TiO_2_ Nanocomposites Reinforced by Electrospun Ceramic Nanofibers

**DOI:** 10.3390/polym13244319

**Published:** 2021-12-09

**Authors:** Hany S. Abdo, Ubair Abdus Samad, Mohamed S. Abdo, Hend I. Alkhammash, Muhammad Omer Aijaz

**Affiliations:** 1 Center of Excellence for Research in Engineering Materials (CEREM), King Saud University, P.O. Box 800, Riyadh 11421, Saudi Arabia; uabdussamad@ksu.edu.sa (U.A.S.); maijaz@ksu.edu.sa (M.O.A.); 2 Mechanical Design and Materials Department, Faculty of Energy Engineering, Aswan University, Aswan 81521, Egypt; 3 Department of Biomedical Engineering, Faculty of Engineering, Minia University, Minia 61519, Egypt; bioengmsa@yahoo.com; 4 Department of Electrical Engineering, College of Engineering, Taif University, P.O. Box 11099, Taif 21944, Saudi Arabia; Khamash.h@tu.edu.sa

**Keywords:** corrosion behavior, ceramic nanofibers, electrospinning, Al metal matrix

## Abstract

This study is focuses on the investigation of the effect of using TiO_2_ short nanofibers as a reinforcement of an Al matrix on the corrosion characteristics of the produced nanocomposites. The TiO_2_ ceramic nanofibers used were synthesized via electrospinning by sol-gel process, then calcinated at a high temperature to evaporate the residual polymers. The fabricated nanocomposites contain 0, 1, 3 and 5 wt.% of synthesized ceramic nanofibers (TiO_2_). Powder mixtures were mixed for 1 h via high-energy ball milling in a vacuum atmosphere before being inductively sintered through a high-frequency induction furnace at 560 °C for 6 min. The microstructure of the fabricated samples was studied by optical microscope and field emission scanning electron microscope (FESEM) before and after corrosion studies. Corrosion behavior of the sintered samples was evaluated by both electrochemical impedance spectroscopy (EIS) and potentiodynamic polarization techniques (PPT) in 3.5% NaCl solution for one hour and 24-h immersion times. The results show that even though the percentage of ceramic nanofibers added negatively control corrosion resistance, it is still possible to increase resistance against corrosion for the fabricated nanocomposite by more than 75% in the longer exposure time periods.

## 1. Introduction

A pure heart is a powerful heart, but a pure material is not enough to endure in tough environments. Scientists all over the world are working hard and continuously to develop more efficient composite materials with better properties by mixing different materials with different phases. Composites consist of two main structures: the base matrix, which is the core element, and the additives or reinforcements embedded in the base matrix [1]. In metal matrix composites (MMC), the base matrix is metal [2], and reinforcements can be any other structure, such as nanoparticles [3], nanotubes [4], nanorods [5] or nanofibers [6,7]. According to Adebisi et al. [8], aluminum is the most common metallic material that can be used as a matrix material, owing to its high strength, acceptable thermal and electrical conductivity, better corrosion and electrochemical behavior [9]. For conventional reinforcement of MMCs, flake and particulate types of ceramic reinforcements are mostly used [10,11,12,13]. Nonetheless, the interference between the metallic matrix and the ceramic reinforcements is generally not perfect, which produces incredibly porous composites with fewer mechanical properties and higher corrosion sensibility [14]. As a way to resolve this problem, nanofibers have been introduced as a novel form of reinforcement in MMCs [15,16,17,18,19,20,21]. The mechanical properties of Al composites are efficiently enhanced in the case of using nanofibers as a reinforcement [20,21] Accordingly, the high surface-to-volume ratio of nanofibers is effectively improved. The strength and stiffness of Al composites is better than that of micro-fibers, due to the good interface between the nano ceramic reinforcement and the metal matrix [6,20]. Electrospun ceramic nanofibers (CNFs) have shown many exceptional features and have been widely implemented in many diverse applications [22,23,24].

Ceramic nanofibers are an brilliant reinforcement material [25] due to their exceptional mechanical and electrochemical properties, such as high strength and elastic modulus, as well as good chemical and thermal stability [26]. TiO_2_ ceramic nanofiber reinforcement, in combination with an Al metal matrix, effectively improves the mechanical properties of the nanocomposite [6]. Research in recent years has been focused on carbon nanofibers and carbon nanotubes as a reinforcement for MMC. Until now, there has been a limited number of studies in which ceramic nanofibers have been successfully introduced in metallic matrix materials resulting in significant improvements in mechanical and electrochemical properties [27,28].

A high-frequency induction-heated sintering (HFIHS) process is one of the most effective consolidation techniques in which simultaneous pressure and temperature are applied to the powder mixture sample in a vacuumed atmosphere within a very short time to produce a high-density and homogeneous composite [29,30]. This advanced sintering process is beneficial because its densification ability is very good, which produces high-density samples with convergent real and theoretical densities, i.e., relative density is very close to 100% [31]. However, extensive research has been conducted in the last few years on MMC reinforced by nanofibers and the development of its mechanical properties. Still, there is a knowledge gap concerning corrosion resistance and electrochemical characteristics of these composites, especially those reinforced by ceramic nanofibers [32,33]. 

Electrospinning produces nanofibers from polymeric material and can produce inorganic nanofibers by combining different inorganic nanoparticles with polymer solutions in order to alter and improve their properties [34,35,36,37]. The combination of electrospinning with other traditional methods has improved the properties of electrospun nanofibers for a wide variety of functional applications. Therefore, in the current study, TiO_2_ ceramic nanofibers were synthesized via electrospinning technique and sol-gel method to be used as a reinforcement for pure a Al matrix. The effect of different reinforcement ratios on the electrochemical characteristics and corrosion behavior of the fabricated nanocomposite in 3.5% NaCl solution was investigated using various electrochemical techniques. Specifically, electrochemical techniques adopted were electrochemical impedance spectroscopy (EIS) and POTENTIODYNAMIC POLARIZATION TECHNIQUES (PPT). Characterization of powder mixture morphology was performed using field emission scanning electron microscopy (FESEM). Characterization of chemical composition was performed using X-ray diffraction (XRD) spectra along the sample’s preparation steps.

## 2. Experimental Procedure

### 2.1. Raw Materials

Aluminum fine powder with 98% purity and an average particle size of 45 μm was purchased from Loba Chemie (Mumbai, India) to be used as base matrix. Polyvinylpyrrolidone (PVP) with a molecular weight of 1,300,000 kg/mole was obtained from Sigma–Aldrich (Burlington, MA, USA), Titanium isopropoxide (C12 H28 O4 Ti). Ethanol (96% purity) was obtained from Avonchem (Macclesfield, Cheshire, UK), and Acetic Acid 99.7% was obtained from Qualikems (Delhi, India).

### 2.2. Ceramic Nanofiber Preparation

**Figure 1:**
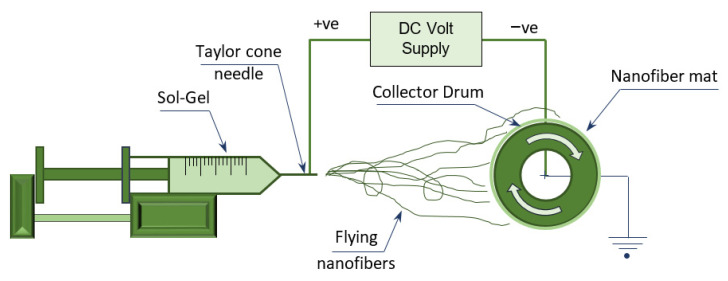
Schematic layout for the electrospinning device employed.

Ceramic nanofibers from TiO_2_ were successfully prepared via sol-gel method by electrospinning of Titanium isopropoxide and PVP (Figure 1), then calcining the produced nanofiber mats in an oxidized environment in order to evaporate the polymer. The mats were then held over the ceramic content of TiO_2_. Sol-Gel was prepared by stirring the solution with gelation for 2–3 h at room temperature in order to produce a clear, transparent, homogeneous mixture. The solution was made by adding 6.75 gm of Ti (IV)-isopropoxide (C12 H28 O4 Ti) to 13.5 mL of acetic acid, while gelation was achieved by adding 2.25 gm of PVP to 45 gm of ethanol.

The prepared sol-gel was poured into a plastic syringe with 20 mL capacity, then loaded on the electrospinning device shown in Figure 1. The electrospinning process was performed using three basic components: high-voltage source (20–22 kV), a syringe with small-diameter needle and a collecting drum of low rotation speed (70–90 rpm).

### 2.3. Calcination Process

In order to convert the polymeric nanofibers prepared by electrospinning to ceramic nanofibers, a calcination process was necessary, in which the green nanofiber mat was burned in air environment at high temperature but below its melting point. The availability of oxygen in the surrounding environment during calcination helped the volatilization reaction to takes place above the thermal decomposition temperature of the burned nanofibers. In the current study, TiO_2_ nanofiber was calcined at 750 °C for 150 min with a heating rate of 12 °C/min using a tube furnace (CARBOLITE Type 3216CC, Chelmsford, Essex, UK).

### 2.4. Composite Preparation

A mixture of Al and TiO2 NF was prepared via high-energy ball milling (HEBM) using a planetary ball mill (Pulverisette 7, Fritsch, Idar-Oberstein, Germany) with zirconium balls and stainless-steel jars. The mixing process was performed with a powder-to-balls ratio of 2:1 wt.% and a speed of 100 rpm for 1 h total milling time (30 min milling + 30 min break + 30 min milling). The percentage of ceramic nanofibers used was 0, 1, 3 and 5 wt.% of the total mixture content. 

### 2.5. Sintering Process (Consolidation)

**Figure 2:**
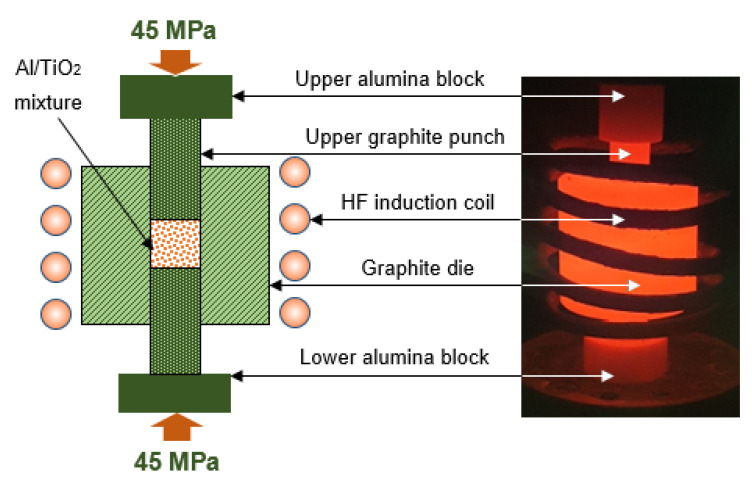
Graphite die setup used for sintering/consolidation process.

The milled powder mixture was inductively sintered using a high-frequency induction heat-sintering furnace (HFIHS Active Sinter System, ELTek Co., Gyeonggi-do, Korea). Three grams of nanocomposite mixture was loaded into a graphite die with 10 mm ID, 35 mm OD and 16 mm height (Figure 2) to produce a cylindrically shaped metal composite sample of 10 mm diameter and 12 mm height per run. Sintering was performed in a vacuumed atmosphere at 560 °C under 45 MPa axial pressure with a theating rate of 200 °C/min and 6 min holding time. Cooling after the consolidation process occurred spontaneously inside the furnace until reaching near room temperature.

### 2.6. Electrochemical Testing and Characterization 

Electrochemical experiments and corrosion studies for the produced samples were performed using a Potentiostat Autolab (PGSTAT302N, Metrohm, Amsterdam, The Netherlands), and the test medium was 3.5% NaCl. A standard three-electrode electrochemical cell accommodating 30 mL of 3.5% NaCl solution was used. In this cell, the produced nanocomposites were used as the working electrode, an Ag/AgCl as reference electrode and a Platinum strip (Pt) as the auxiliary or counter electrodes. The EIS data were obtained at the open-circuit potential value, with frequencies ranging from 100 MHz to 100 mHz, by applying a −5 mV amplitude sinusoidal wave perturbation at the corrosion potential (E_Corr_). The cyclic potentiodynamic polarization (CPP) experiments were carried out by scanning the potential between −1600 mV and +100 mV (Ag/AgCl) with a scanning rate of 1.5 mV/s at room temperature. The tested surface area of all samples was the same and equal to π/4 (10 mm)^2^ ≅ 78.5 mm^2^. All samples were cleaned with acetone, then washed by distilled water and dried by air after being polished with emery paper and cloth-polished by alumina slurries before every test. Each test was repeated at least 3 times to ensure repeatability. 

SEM micrographs and EDX investigations were conducted using a JEOL field emission scanning electron microscopy (FESEM) (model: JEOL JSM-7600F, Tokyo, Japan) with an energy-dispersive X-ray spectroscopy (EDS) unit from Oxford instruments attached. The chemical composition of the produced consolidated Al/TiO^2^ nanocomposite samples was obtained using an X-Ray diffraction pattern (XRD) (model: D8 discover from Bruker, Germany) with filtered Cu Kα radiation (λ = 1.5406 Å).

## 3. Results and Discussion

### 3.1. Ball-Milled Powder Morphology

**Figure 3:**
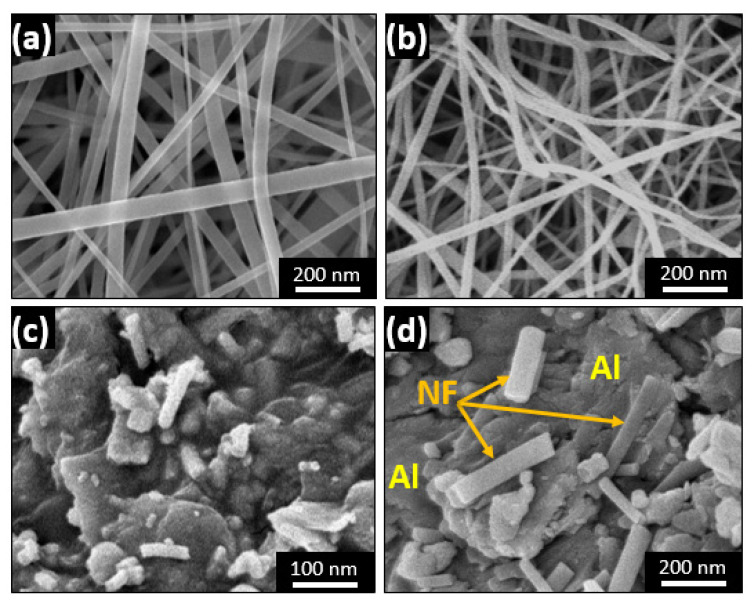
SEM images for (**a**) PVP/TiO_2_ nanofiber mat before calcination, (**b**) TiO_2_ nanofiber after calcination, (**c**,**d**) Al/TiO_2_ powder mixture at different magnifications.

The first and second steps of ceramic nanofiber reinforcement preparation is electrospinning of PVP/TiO_2_ sol-gel, followed by the calcination process. Figure 3a,b illustrate the produced nanofiber mat after electrospinning and after calcination, respectively. The average fiber diameter range is about 50–110 nm, with homogeneous and uniform structure and no defects. The change in nanofiber morphology following calcination consists mainly of some reduction in the diameter, with little distortion due to the evaporation of carbon during the high-temperature calcination process.

The morphology of the produced mixed powder of Al as the base matrix and the amount of the fabricated reinforcement of TiO_2_ ceramic nanofibers and their distribution can affect the electrochemical properties of the composites. Figure 3c,d illustrate the morphology of the mixture in the case of 5 wt.% ceramic nanofiber used in an Al matrix after complete mixing by ball milling for 1 h.

**Figure 4:**
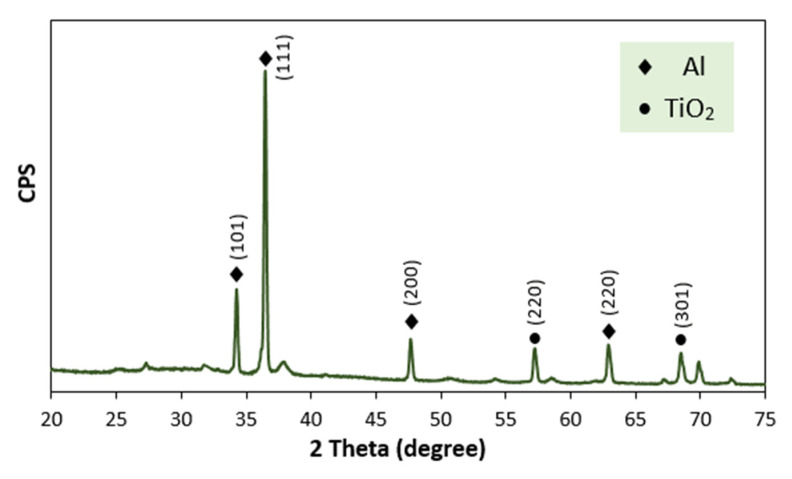
XRD of the 5 wt.% Al/TiO_2_ consolidated sample.

The chemical composition of the consolidated ball-milled mixed powders was investigated by XRD and is presented in Figure 4. The XRD diffraction peaks for the composite material Al/TiO_2_ is clear, and main peaks correspond to Al peaks, which are present at 2θ = 34, 37 and 62°, as long as the TiO_2_ nanofiber main peaks are at approximately 2θ = 57 and 68°.

### 3.2. Electrochemical Measurements

#### 3.2.1. Electrochemical Impedance Spectroscopy (EIS)

**Figure 5:**
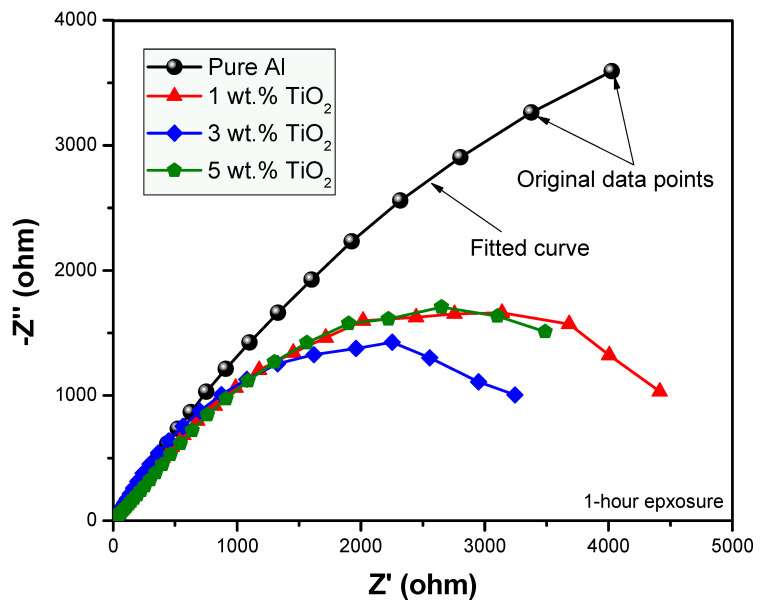
EIS for composite samples exposed for 1-h in 3.5% NaCl solution.

**Figure 6:**
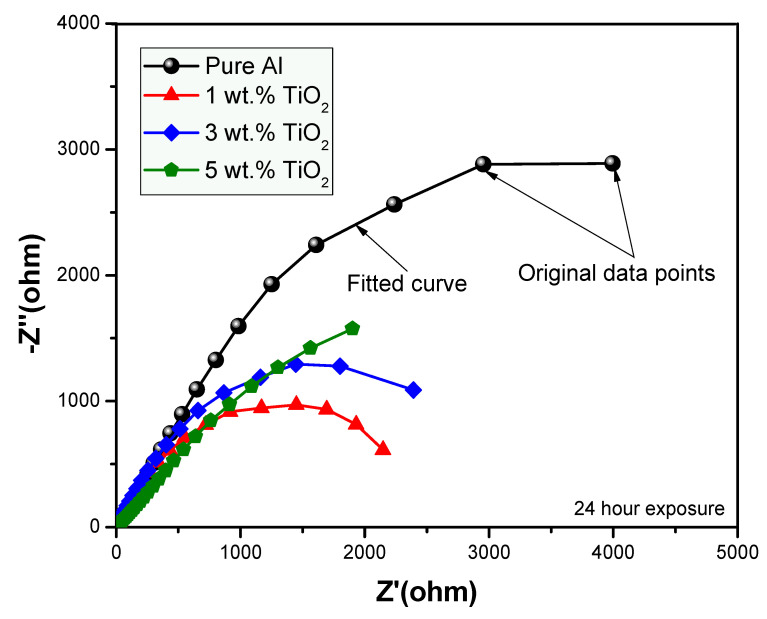
EIS for composite samples exposed for 24-h in 3.5% NaCl solution.

**Figure 7:**
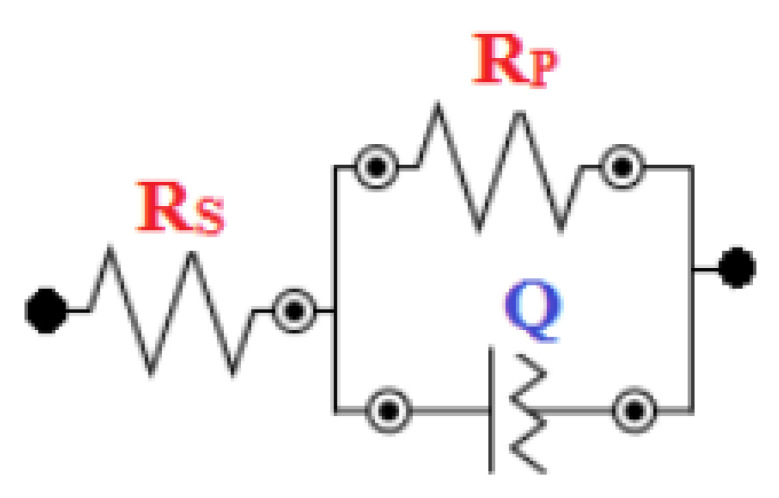
Equivalent circuit used for Nyquist plot analysis.

In order to determine the anticorrosion properties of the prepared nanocomposites exposed to the aggressive environment of 3.5% NaCl solution, the EIS technique was employed [38,39,40,41,42]. Nyquist plots were obtained for the fabricated samples with compositions of 0, 1, 3 and 5 wt.% by varying their percentages for the immersion time of 1 h, as shown in Figure 5. The Nyquist plot obtained for the prolonged exposure period of 24 h for the same samples is shown in Figure 6. The fitting circuit used to fit the obtained graphs is shown in Figure 7, where Rs is the solution resistance; Rp is the polarization resistance, which can also be defined as charge-transfer resistance; and Q is the constant phase element (CPE). The results obtained by applying this circuit to fit are presented in Table 1.

**Figure 8:**
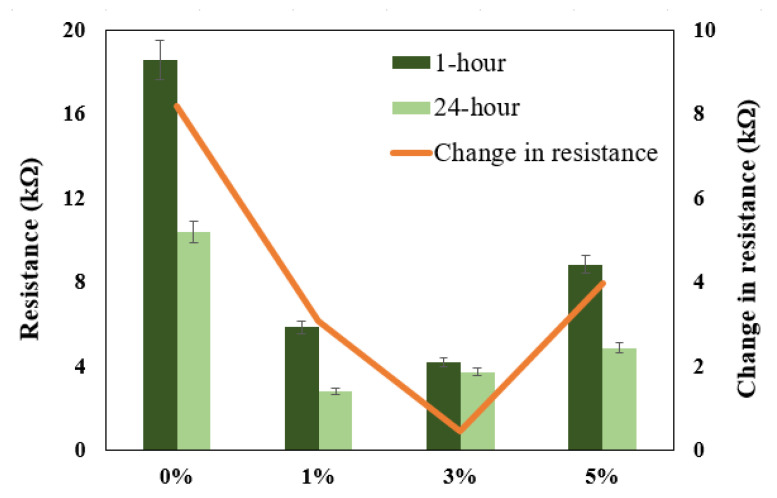
Graphical representation of Rct obtained after 1 h and 24 h exposure to NaCl solution.

It can be seen from Figure 5 and Figure 6, with exposure periods of 1 h and 24 h immersion results in 3.5% NaCl solution, that for all samples, there is only one distorted semicircle. With the increasing amount of TiO_2_ ceramic nanofibers as a reinforcement in the Al matrix, the semicircle becomes more depressed, and the diameter of the semicircle also decreases, which suggests that at the lower exposure time of 1-h, the prepared alloys have lower resistance to corrosion. It is generally agreed that the wider the diameter of the semicircle, the higher the corrosion resistance. The data obtained with the fitting circuit are shown in Table 1. The Rct value for all the alloys decreased with the inclusion of ceramic nanofibers, although the highest value of Rct was obtained with 5% inclusion when compared to all the prepared alloys, though still lower than the control sample without any ceramic fibers. The value of “*n*” in the CPE is in the range of 0.63 to 0.77 for the tests conducted on alloys, which represents CPE behaving like capacitance when the values of “*n*” happen to be in between 0 and 1. On the other hand, *n* = 0 represents pure resistance, *n* = 1 represents pure capacitance and *n* = 0.5 represents Warburg. In the case of our analysis, the results indicate that the surface is affected because of its exposure to NaCl solution. The values obtained for CPE decreased with the addition of ceramic nanofibers. With the increase in exposure time to 24-h, the results indicate a further decrease in diameter of all samples, which is due to the corrosion occurring on the alloy surface. Resistance against corrosion was found to decrease with the incorporation of ceramic TiO_2_ nanofibers, as the percentage of corrosion increased in comparison to the sample with no fibers. Figure 8 shows the resistances at exposure periods of both 1 h and 24 h in order to get an idea concerning the corrosion of alloys after the addition of ceramic fibers. It can be concluded from Figure 8 that addition of ceramic fibers deteriorates the anticorrosion properties of the prepared alloys, with even small-percentage changes making it more prone to corrosion. Although the difference between 1 h and 24 h resistance (Rct) for alloys prepared with 3% fibers is not significant compared to alloys, it it is still lower than for samples without any fibers. This is because Al undergoes corrosion with the formation of an oxide layer, which acts as passivation, therefore blocking further penetration of corrosive species. On the other hand, the addition of ceramic fibers to an Al matrix creates voids on the exposure surface, which have to be considered while taking up the surface area of the Al composite. These fiber-metal boundaries act as potential sites of attack by corrosive species, thus causing localized corrosion and making the material more prone to corrosion, which, in our case, resulted in deterioration of corrosion properties.

#### 3.2.2. Cyclic Potentiodynamic Polarization (CPP)

**Figure 9:**
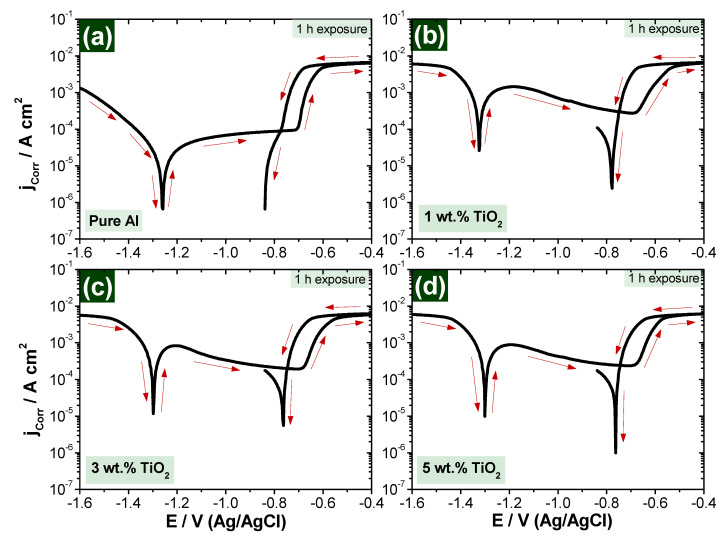
CPP for composite samples exposed for 1-h in 3.5% NaCl solution (**a**) Pure Al, (**b**) 1 wt.% TiO_2_, (**c**) 3 wt.% TiO_2_ and (**d**) 5 wt.% TiO_2_.

**Figure 10:**
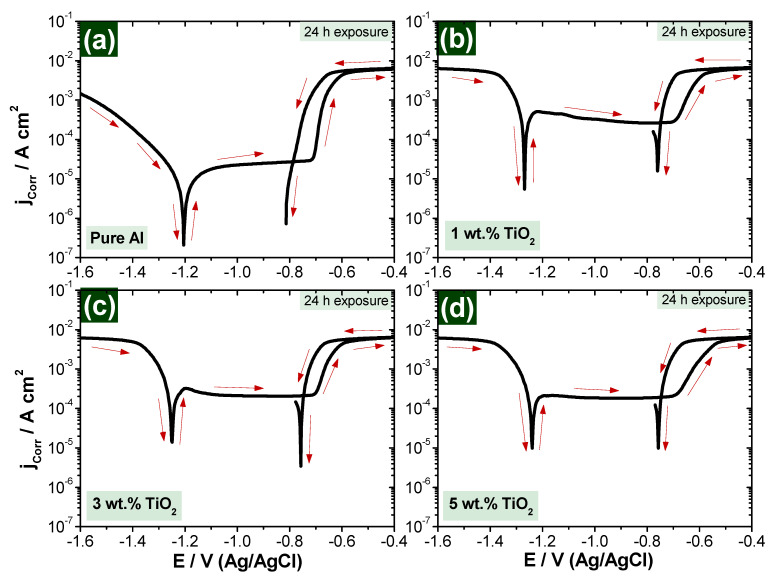
CPP for composite samples exposed for 24-h in 3.5% NaCl solution (**a**) Pure Al, (**b**) 1 wt.% TiO_2_, (**c**) 3 wt.% TiO_2_ and (**d**) 5 wt.% TiO_2_.

The curves of Figure 9 and Figure 10 present the CPP measurements for the fabricated nanocomposite samples after immersion in 3.5% NaCl solution for 1 and 24 h, respectively. Table 2 is presents all values of the extracted parameters from CPP plots, such as polarization resistance (R_P_), corrosion rate (R_Corr_), corrosion current density (j_Corr_), corrosion potential (E_Corr_) and anodic and cathodic Tafel slopes (β_a_ and β_c_). All parameters, including the corrosion rate, were calculated automatically by Autolab software (NOVA). Tafel slope was used to extract icorr and Ecorr from polarization data. The values were extracted by drawing anodic and cathodic slopes in the NOVA software, which then automatically calculated the values of icorr and Ecorr.

It is clearly shown from Figure 9 and Figure 10 that scanning the potential in the less negative direction leads to a decrease in the produced current in the cathodic portion due to the decrease in the rate of cathodic reaction of oxygen reduction at the more positive potentials [43,44,45], which helps to start the reaction of the cathode, followed by adsorption, according to Equation (1):

(1) $$\frac{1}{2}O_{2}+H_{2}O+2e^{-}=2{\mathrm{OH}}^{-}$$

In anodic reaction, the corrosion takes place due to the dissolution reaction once the corrosive medium becomes available. In our case, Al is the active material to start the dissolution reaction on the sample surface, forming aluminum oxide (Al_2_O_3_) [46,47,48,49] and causing an increase in the anodic current [43,44,45], according to Equation (2):

(2) $$\mathrm{Al}\;\to {\mathrm{Al}}^{3+}+3e^{-}$$

According to Equation (3), the rate of increase of the current in the anodic portion is slowed down by the application of the potential towards the less negative values due to the formation of oxide film Al_2_O_3_ [45] as follow:

(3) $$3{\mathrm{OH}}^{-}+2{\mathrm{Al}}_{\mathrm{surface}}={\left[{\mathrm{Al}}_{2}O_{3}\right]}_{\mathrm{adsorb}}+3e^{-}$$

Passivation of the fabricated nanocomposite sample surface is increased by increasing the reinforcement material, which is TiO_2_ in our study. The presence of TiO_2_ increases the passive region on the curves of the polarization measurements due to the reduction in the current values.

**Figure 11:**
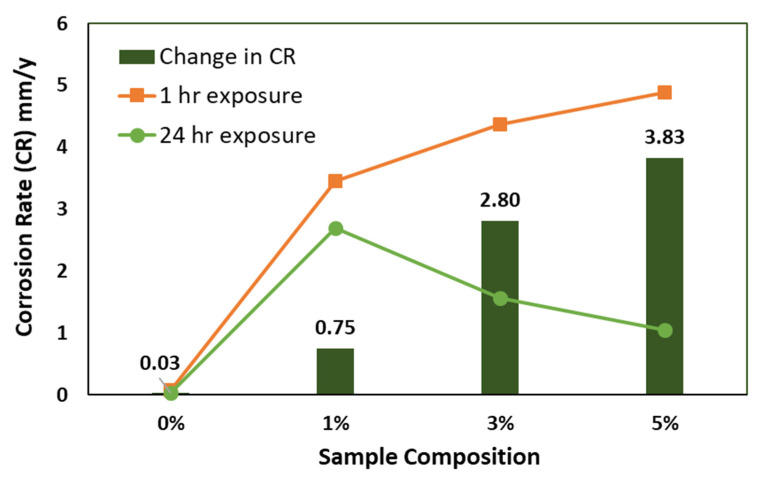
Graphical representation of corrosion rate (CR) after 1 h and 24 h exposure to NaCl solution.

Polarization curves (Figure 9 and Figure 10) indicate that the change in corrosion rate between the immersion time of 1 h and 24 h samples in 3.5% NaCl solutions is decreased positively by increasing the reinforcement percentage, as presented in Figure 11. 

From polarization curves, which are presented in Figure 9 and Figure 10, it can be concluded that the addition of ceramic nanofibers can increase resistance against corrosion in longer exposure periods, which is in agreement with the conclusion based on the impedance curves in Figure 5 and Figure 6.

## 4. Conclusions

Fabrication of Al/TiO_2_ nanocomposite reinforced by different percentages of ceramic nanofibers was achieved using powder metallurgy. Ceramic nanofibers were produced via electrospinning technique, followed by calcination at 750 °C. Al powder plus ceramic nanofibers were mixed together with different compositions using a high-energy ball milling technique at 100 rpm for 1 h. The homogeneous mixture was consolidated through an inductive sintering process at 560 °C and 45 MPa axial pressure with a heating rate of 200 °C/min and 6 min holding time. The fabricated nanocomposite samples were characterized, then electrochemically tested against corrosion. The effect of increasing the amount of ceramic nanofiber reinforcement from 0 wt.% up to 5 wt.% on corrosion behavior after 1 h and 24 h immersion in 3.5% NaCl solutions was reported. The investigations were carried out using different electrochemical techniques, namely electrochemical impedance spectroscopy and cyclic potentiodynamic polarization, along with characterization by methods such as FE-SEM and XRD. Electrochemical measurement results confirm that the addition of ceramic nanofibers to an Al matrix negatively affects its resistance against corrosion. On the other hand, the addition of ceramic nanofibers can increase resistance against corrosion for the same fabricated nanocomposite in longer exposure time periods. In a quantitative description, the enhancement of corrosion resistance for the 3 and 5 wt.% TiO_2_ reinforced sample can achieve to 65% and 75% amelioration, respectively by increasing the immersion period from 1 h to 24 h in 3.5% NaCl solution.

## Figures and Tables

**Table 1:** Obtained parameters after EIS experiment on the composite samples with exposures of 1 h and 24 h in 3.5% NaCl solutions.

Sample	Time	Rct (k.ohm)	Q	
			Y0 (µMho)	*n*
Pure Al	1-h	18,600	204	0.63
1 wt.% TiO_2_		5870	70.1	0.65
3 wt.% TiO_2_		4170	109	0.73
5 wt.% TiO_2_		8860	180	0.67
Pure Al	24-h	10,400	203	0.71
1 wt.% TiO_2_		2790	118	0.77
3 wt.% TiO_2_		3730	184	0.70
5 wt.% TiO_2_		4870	250	0.69

**Table 2:** Obtained parameters of the composite samples from CPP plots with exposures of 1-h and 24-h in 3.5% NaCl solutions.

Sample		Parameter					
		βa/mV·dec^−1^	βc/mV·dec^−1^	E_Corr_/V	j_Corr_/µA·cm^−2^	R_p_/kΩ·cm^2^	R_Corr_/mmpy
1 h	Pure Al	0.074007	0.059014	−1.2594	5.73	2.4901	0.06654
	1 wt.% TiO_2_	0.10686	0.056606	−1.3231	296.75	0.054155	3.4482
	3 wt.% TiO_2_	0.20704	0.10647	−1.2967	375.90	0.081233	4.3679
	5 wt.% TiO_2_	0.23471	0.10866	−1.2992	419.99	0.076807	4.8803
24 h	Pure Al	0.066761	0.07771	−1.2038	3.12	4.992	0.036303
	1 wt.% TiO_2_	0.10459	0.057999	−1.2683	232.21	0.069779	2.6983
	3 wt.% TiO_2_	0.052622	0.1107	−1.2497	134.54	0.11514	1.5633
	5 wt.% TiO_2_	0.051207	0.057243	−1.2396	90.73	0.12938	1.0542

## Data Availability

Not applicable.
